# Structure–Property Relationships in PVDF/SrTiO_3_/CNT Nanocomposites for Optoelectronic and Solar Cell Applications

**DOI:** 10.3390/polym16060736

**Published:** 2024-03-07

**Authors:** Taha Abdel Mohaymen Taha, Sultan Saud Alanazi, Karam S. El-Nasser, Alhulw H. Alshammari, Ali Ismael

**Affiliations:** 1 Physics Department, College of Science, Jouf University, P.O. Box 2014, Sakaka 72388, Saudi Arabia; 2 Department of Chemistry, College of Science and Arts, Jouf University, Sakaka 72388, Saudi Arabia; 3 Physics Department, Lancaster University, Lancaster LA1 4YB, UK

**Keywords:** PVDF polymer, SrTiO_3_, CNTS, optical band gap, refractive index

## Abstract

The optical properties of polyvinylidene fluoride (PVDF) polymer nanocomposite films incorporating SrTiO_3_/carbon nanotubes (CNTs) as nanofillers are investigated. PVDF/SrTiO_3_/CNTs films were prepared by the solution casting technique. X-ray diffraction (XRD), Fourier-transform infrared spectroscopy (FTIR), and scanning electron microscopy (SEM) analyses confirmed the incorporation of SrTiO_3_/CNTs into the PVDF matrix. The addition of nanofillers influenced the crystalline structure, morphology, and optical properties of the films. SEM images showed spherulite morphology, which is a spherical aggregate of crystalline polymer chains. The addition of a SrTiO_3_/CNTs nanofiller modified the polymer’s electronic structure, causing a variation in the energy gap. The addition of SrTiO_3_/CNTs at 0.1 wt% increased the band gap, refractive index, and nonlinear optical properties of the PVDF films. These improvements indicate the potential of these nanocomposite films in optoelectronic applications such as solar cells, image sensors, and organic light-emitting diodes.

## 1. Introduction

Polymers are crucial for advancements in the electronics, communication, and energy sectors, enabling lighter, more efficient devices [1,2,3]. The key characteristic of a polymer nanocomposite is the presence of nanofillers dispersed within the polymer matrix. Polymer nanocomposites offer superior properties compared to traditional polymers due to their distinct properties that qualify their applications in nanotechnology and biotechnology. Suitable fillers can improve the physical or chemical properties of the nanocomposites [4,5,6]. To end up with very good nanocomposites, there are many factors that must be carefully considered. For instance, the homogenous dispersion of the fillers, i.e., nanoparticles in the polymer matrix, is expected to enhance their properties. Factors like the nature of the polymer and nanoparticle, particle size, and concentration can significantly impact the desired function of nanoparticles within the matrix [7]. Accordingly, polymer nanocomposites find diverse applications in industries including solar cells, energy storage, sensors, optoelectronics, and water desalination [8].

Polyvinylidene fluoride (PVDF) is a semicrystalline polymer that contains the repeated monomer unit CH_2_=CF_2_. The PVDF comes in various forms, such as (α, β, γ, δ, and ε). These forms differ in the arrangement of their molecular chains, which creates a permanent electrical polarity (dipole moment) perpendicular to the chain direction [9,10,11,12]. The PVDF phase is important to allocate to the appropriate application; for instance, its crystallized β phase has a good chance of being used in piezoelectric applications. In addition, the crystallized b phase can be enhanced due to the implementation of ferrite nanomaterials in the PVDF. In general, the properties of PVDF are affected by the spatially symmetrical disposition of hydrogen and fluorine atoms [13,14]. PVDF polymer nanocomposites are rapidly gaining prominence in both industrial and scientific applications. [15,16]. PVDF polymer nanocomposites have excellent electrical, optical, and mechanical properties. PVDF–ferrite nanocomposites combine magnetic and electric properties [17,18]. PVDF nanocomposite films have good optical properties, which can be controlled through various factors such as the homogeneity of the matrix, the concentration and size of the particles in the matrix, and preparation methods. Therefore, adjusting these factors to the optimal level increases the high-quality production films of PVDF nanocomposites. Because the PVDF polymer is transparent, the inclusion of nanoparticles in the PVDF polymer matrix is a sensitive process since it may affect its degree of transparency [19]. The PVDF matrix film can be doped with nanoparticles, for example, quantum dots or carbon, to obtain composite active films that are used in energy conversion [20]. Here, nanoparticles play a major role in absorbing light within a wide wavelength range and then transferring its energy to the PVDF matrix to be converted into electrical energy. Adjecting the carbon nanotube concentration into the PVDF film would enhance the energy conversion efficiency [21].

Strontium titanate (SrTiO_3_) is a perovskite oxide with a cubic structure at room temperature and a wide band gap energy of 3.2 eV. It is characterized by a low value of dielectric loss and leakage current as well as a high dielectric constant [22,23]. Incorporating SrTiO_3_ into a polymer matrix enhances its thermal and optical properties. Carbon nanotubes (CNTs) scatter light to increase photon path length, enhancing light absorption, specifically in the UV and visible ranges. SrTiO_3_ nanoparticles also aid in light absorption as plasmonic scatterers. Both CNTs and SrTiO_3_ improve the thermal stability of the polymer, which is critical for solar cell applications to prevent degradation and reduce power conversion efficiency.

Theoretical studies of polymer nanocomposite films delve into understanding how the incorporation of nanoparticles into a polymer matrix affects their optical and band structure properties. These studies employ various computational techniques and models to predict and explain the observed behavior in these materials. Density functional theory (DFT) simulates the electronic structure of the entire system, including the polymer matrix, nanoparticles, and their interface [24]. It can predict band gaps, electronic density distribution, and optical absorption spectra. In one study, the band gap of polyaniline (PANI) was calculated using the B3LYP functional and SV(P) basis set, resulting in a band gap of 1.9 eV for a commonly used structural motif [25]. A machine learning model called support vector regression (SVR) was developed to predict polymer band gaps, achieving a high determination coefficient (R2) of 0.824 for leave-one-out cross-validation [26]. Lastly, a new polymer acceptor made from naphthalenediimide (NDI) and bifuran units exhibited an ultranarrow optical bandgap of 1.26 eV and improved light-absorbing properties [27]. Tight-binding models capture the essential interactions between atoms or molecules, allowing for efficient calculations of band structures and electronic properties [28]. Effective medium theories treat the nanocomposite as a homogeneous material with averaged properties based on the individual components and their volume fractions [29]. 

Polymers have distinct optical properties like transparency and refractive index, which are crucial for various applications. These properties can be optimized by factors like blend homogeneity and particle size. The nanocomposites of PVDF–graphene oxide (GO) were prepared so that the addition of GO increased the absorbance and affected the refractive index [23]. PVDF/ZnO nanocomposite films were synthesized, and as the weight of ZnO increased in the films, the refractive index increased, while the direct and indirect energy gap decreased [30]. PVDF/MgCl_2_ nanocomposite films were prepared, and their optical properties were investigated in relation to the MgCl_2_ content. The addition of MgCl_2_ improved the optical energy gap of the film [31]. El-Masry and Ramadan [32] have reported the effect of nanoparticles such as CoFe_2_O_4_, CuFe_2_O_4_, and Cu/CoFe_2_O_4_ on the optical properties of PVDF polymer nanocomposites. The nanoparticles enhanced the refractive index of the PVDF nanocomposites, and the polarizability was enhanced upon the addition of CuFe_2_O_4_. A previous study showed that the refractive index of the PVDF polymer nanocomposites can be influenced by the inclusion of Ag nanoparticles. In addition to that, the light absorption in the visible region has increased [33]. The effect of titanium dioxide (TiO_2_) on the optical properties of a PVDF matrix has been studied [34]. The incorporation of TiO_2_ nanoparticles increased the refractive index, improved the optical transparency, and aided the PVDF nanocomposites to selectively absorb within the ultraviolet (UV) spectrum. PVDF–CaFe_2_O_4_ polymer films were synthesized, and their optical properties were investigated. Here, the improvement of the energy gap, refractive index, and optical susceptibility due to the inclusion of CaFe_2_O_4_ into PVDF polymer films was studied, as reported in [35]. In the literature [36], there are studies on the effect of strontium titanate (SrTiO_3_) as a dopant on the properties of polymer film nanocomposites. Taha and Alzara investigated the influence of the inclusion of SrTiO_3_ on the structural, thermal, and dielectric properties of polyvinyl alcohol (PVA) nanocomposite films. The incorporation of different contents of SrTiO_3_ into the PVC/PVP polymer blend has been studied, as reported in [36]. The study showed the impact of SrTiO_3_ additions on optical properties such as optical absorption, linear and nonlinear refractive index, and optical susceptibility of PVC/PVP polymer blend. 

This work investigates the novel combination of strontium titanate (SrTiO_3_) and carbon nanotubes (CNTs) as nanofillers within polyvinylidene fluoride (PVDF) polymer nanocomposite films. The synergistic effects of these nanofillers on the optical properties of the nanocomposites have not been previously explored, offering new insights into the design of functional materials. Therefore, PVDF/SrTiO_3_/CNTs films were prepared by the solution casting technique. XRD, FTIR, and SEM analyses confirmed the incorporation of SrTiO_3_/CNTs into the PVDF matrix. The optical band gap of PVDF polymer films increased after incorporating 0.1 wt% SrTiO_3_/CNT nanofiller. At higher doping levels, the concentration of SrTiO_3_/CNT at the interface with PVDF could increase significantly, effectively narrowing the band gap. The estimated values of static refractive index (n_0_) showed an increase (2.42–4.27) upon increasing the percentage of SrTiO_3_/CNTs nanofiller. The addition of SrTiO_3_/CNTs nanofillers increases the polarizability of the polymer molecules and, hence, the nonlinear refractive index.

## 2. Experimental Details

### 2.1. Materials

Polyvinylidene fluoride (PVDF) powder was supplied by Alfa Aesar (Haver Hill, MA, USA, strontium titanate), (SrTiO_3_) nanopowder and carbon nanotubes (CNTs) were supplied by Nanografi (Ankara, Turkey), and ethanol absolute and dimethylformamide (DMF) were supplied by Sigma Aldrich (St. Louis, MO, USA). 

### 2.2. Preparation of SrTiO_3_/CNTs Nanocomposite

A total of 0.8 g of SrTiO_3_ nanopowder and 0.2 g of carbon nanotubes were mixed in 50 mL of ethanol for 60 min. After that, the mixture was subjected to ultrasonic waves for 60 min. The mixture was then dried at 50 °C inside an electric oven. Finally, the product was ground and stored for further investigation.

### 2.3. Preparation of PVDF/SrTiO_3_/CNTs Polymer Films

A total of 1.0 g of PVDF was dissolved in 30 mL of dimethylformamide (DMF) using magnetic stirring at 60 °C for 1 h. SrTiO_3_/CNTs were added to the clear PVDF solution in small amounts: 0.001, 0.003, and 0.007 g. The samples were labeled as P_0_ for pure PVDF and P_1_, P_3_, and P_7_ for nanocomposite films. The mixture was stirred regularly for another 1 h after each addition. The mixture was then transferred to a glass Petri dish. The PVDF/SrTiO_3_/CNTs composite was air-dried at 80 °C. After drying, the composite formed a sheet with a thickness of around 200 μm. This sheet was then peeled off the glass plate.

### 2.4. Characterization Techniques

Phase analysis is a crucial step in qualitative analysis, wherein the identification of phase type, phase composition, crystallite size, and orientation are among the pivotal data points collected. X-ray diffraction patterns for polymer films were generated by Shimadzu XRD 700 (Shimadzu, Kyoto, Japan) with Cuk_α_ wavelength. A Shimadzu 100-FTIR tracer was used to perform FTIR analysis using the ATR technique in the mid-infrared region (400–4000 cm^−1^). The Quatro environmental scanning electron microscope (ESEM) (Thermo Fisher Scientific, Waltham, MA, USA) was used to conduct a microstructural analysis of polymer films. Prior to imaging, the films were sputter-coated with a thin layer of gold to enhance conductivity. Energy-dispersive X-ray spectroscopy (EDS) with an Oxford instruments ISIS unit (Oxford instruments, Abingdon, UK) was used to map the elemental composition of the P7 polymer sample. Operating parameters were 20 kV acceleration voltage and 10 mm working distance. UV-visible absorption spectra of polymer films were generated by a Thermo Scientific Evo 201 spectrophotometer (Thermo Fisher Scientific, Waltham, MA, USA).

## 3. Results and Discussion

### 3.1. X-ray Diffraction Analysis

**Figure 1:**
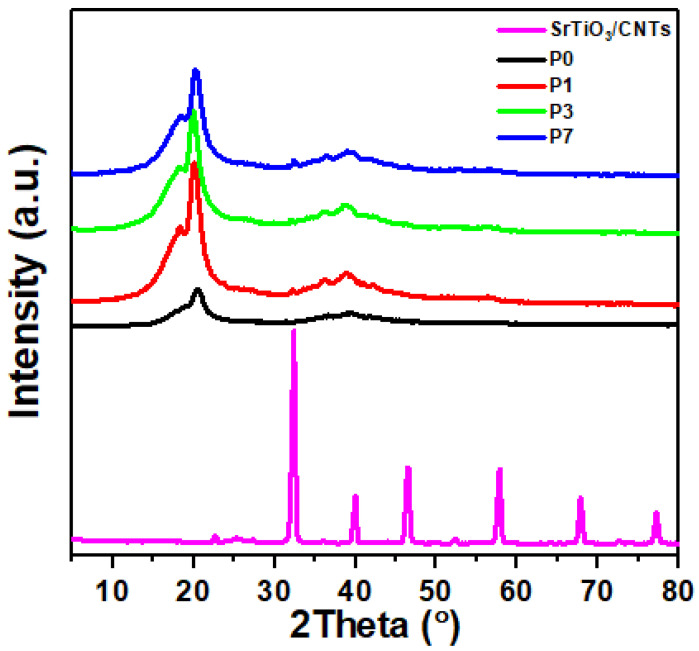
XRD diffraction peaks for PVDF/SrTiO_3_/CNT polymer films.

Figure 1 shows the XRD patterns for the samples of PVDF, SrTiO_3_/CNT, and PVDF with different contents of SrTiO_3_/CNT. For the SrTiO_3_/CNT sample, the diffraction peaks that appeared at 2θ = 32.37°, 39.9°, 46.4°, 57.7°, 67.8°, and 77.1° correspond to the (110), (111), (200), (211), (220) and (310) planes of SrTiO_3_, respectively [37]. For the pure PVDF sample, the diffraction peaks located at 2θ = 20.17° and 39.4° are related to the α phase of the (110) and (002) planes, respectively [35,38]. Moreover, the peak located at 26.6° is also due to the α phase of PVDF [39]. The diffraction peak intensity located at 39.4° decreases after the inclusion of SrTiO_3_/CNT. In addition, the main two peaks located at 2θ = 20.02° and 38.9° for the PVDF sample containing 0.1 wt% SrTiO_3_/CNT shift to 2θ = 20.26° and 39°, respectively, when increasing the content of SrTiO_3_/CNT to 0.3 wt%. The observed shifts arise from specific interactions between the PVDF, SrTiO_3_, and CNTs at the interface. These interactions could involve charge transfer, hydrogen bonding, or other physical/chemical processes that modify the local atomic environment around the filler particles. These results demonstrate the successful preparation of polymer nanocomposites.

The Debye–Scherrer equation (Equation (1)) is a widely used method for estimating the crystallite size [40,41]. Therefore, it was applied to calculate the crystal size of SrTiO_3_. It was found that the SrTiO_3_ average crystallite size is 15.17 nm.

(1) $$D=\frac{0.9\lambda }{\beta cos\theta }$$

where D and λ represent the crystallite size and X-ray wavelength, respectively, while β represents the full width at half maximum. PVDF typically has a semi-crystalline structure with tightly packed chains. Introducing SrTiO_3_ nanoparticles might lead to limited space for growth within the polymer matrix. Additionally, the PVDF chains themselves might not offer sufficient nucleation sites for the controlled growth of SrTiO_3_ crystals.

### 3.2. FTIR Spectroscopy

**Figure 2:**
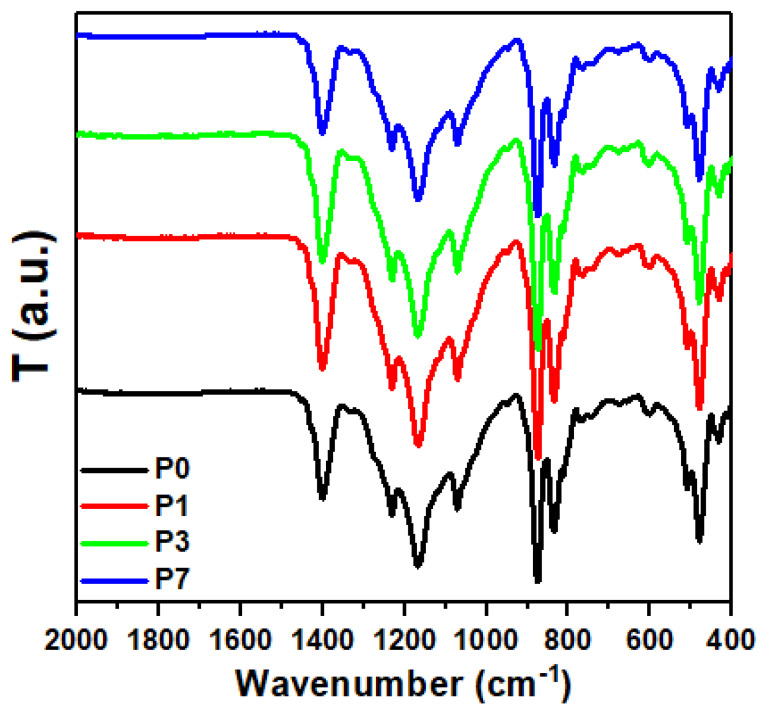
FTIR spectra for the samples of PVDF/SrTiO_3_/CNTs films.

Figure 2 displays the FTIR absorption bands for PVDF, SrTiO_3_/CNT, and PVDF with different contents of SrTiO_3_/CNT. The PVDF β-phase presents the characteristic bands located at 1230, 1167, 1070, 873, 838, 509, 480, and 423 cm^−1^, while bands at 614, 763, and 1400 cm^−1^ are related to the α phase [35,42]. The vibration of the CF_2_ group in PVDF generated the absorption band at 614 cm^−1^, while the band at 763 cm^−1^ was attributed to rocking vibration. The stretching vibrations of C-F caused the absorption band to be cantered at 1400 cm^−1^ [43].

The stretching of C-C and the wagging of H-C-H generated the absorption bands cantered at 1070 and 1167 cm^−1^, respectively. The rocking vibrations of H-C-H generated the band located at 1230 cm^−1^ [44]. Moreover, the band located at 873 cm^−1^ is due to CH_2_ rocking vibrations and CF_2_ stretching vibrations [45]. The rocking mode of H-C-H and the stretching mode of F-C-F generated the bands at 838 cm^−1^, while the bending vibration of F-C-F generated the bands at 480 and 509 cm^−1^ [46]. The absorption bands located at 413 cm^−1^ and 451 cm^−1^ for the pure PVDF sample have shifted to a lower wavenumber upon the addition of SrTiO_3_/CNT. This indicates the successful formation of PVDF/SrTiO_3_/CNT.

The shifts arise from specific interactions between the PVDF chains and the SrTiO_3_/CNT filler at the interface. These interactions might involve hydrogen bonding, charge transfer, or electrostatic forces that alter the electron density distribution around the functional groups responsible for the FTIR bands.

### 3.3. Scanning Electron Microscopy (SEM)

**Figure 3:**
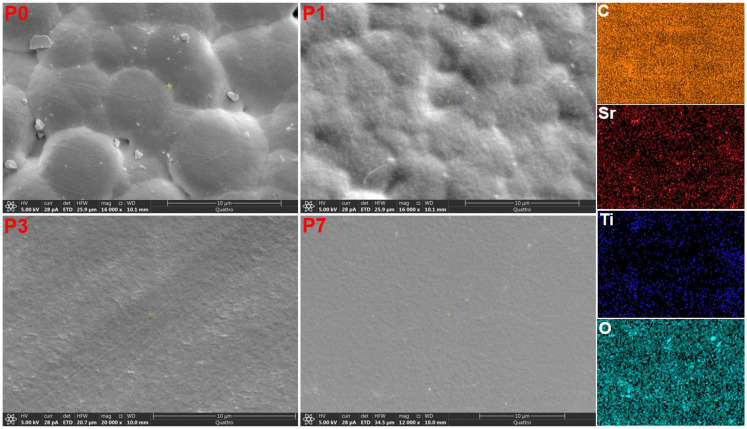
The ESEM micrographs for the PVDF/SrTiO_3_/CNTs films and EDS elemental mapping of the sample P7.

The surface morphology scans of PVDF/SrTiO_3_/CNTs polymer films are displayed in Figure 3. A spherulite morphology was observed, which is a spherical aggregate of crystalline PVDF polymer chains. It is the characteristic morphology of many semi-crystalline polymers, including PVDF. Spherulites are formed by the growth of polymer crystals at a nucleation site. The crystals grow radially from the nucleation site, forming a spherical shape.

The size and shape of the spherulites were affected by the doping rate of the SrTiO_3_/CNT nanofiller. The size of spherulites decreased upon increasing the content of the nanofiller. SrTiO_3_/CNTs can act as nucleation sites for spherulite growth in the PVDF matrix. However, the concentration and distribution of these nucleation sites can vary with the doping rate. Higher doping might lead to an increased number of nucleation sites, resulting in smaller spherulites due to competition for crystallizing molecules. Alternatively, if the filler disrupts the polymer chain mobility or hinders efficient growth, it could lead to smaller or irregularly shaped spherulites. Moreover, interactions between the PVDF chains and the SrTiO_3_/CNTs at the spherulite boundary can influence growth patterns and morphology. These interactions could involve hydrogen bonding, charge transfer, or steric hindrance, potentially modifying the crystal packing and orientation within the spherulites. The elemental mapping of C, Sr, Ti, and O for the sample P_7_ shown in Figure 3 indicates the homogeneous distribution of nanofiller.

### 3.4. UV-Vis Optical Absorption Spectroscopy

**Figure 4:**
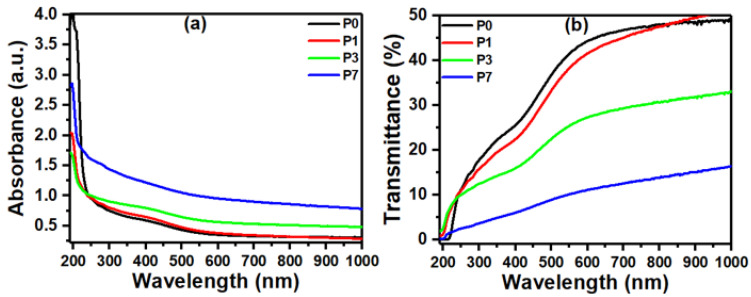
The plots of (**a**) absorbance and (**b**) transmittance versus wavelength data for the PVDF/SrTiO_3_/CNTs films.

Studying polymer films through their optical absorption spectra offers valuable insights into their properties. These spectra reveal key optical characteristics like absorption and transmission, along with the crucial parameters of absorption coefficient and energy gap [47]. By analyzing these features, scientists gain a deeper understanding of the electronic structure and energy levels within the polymers [48]. The absorbance values for the PVDF/SrTiO_3_/CNTs films in the 200–1000 nm spectral bandwidth are displayed in Figure 4a. PVDF chains contain conjugated double bonds formed by alternating carbon-carbon single and double bonds. These double bonds can undergo π-π* electronic transitions when excited by UV light at around 280 nm, as shown in Figure 4 [49].

Interactions between the PVDF and the SrTiO_3_/CNTs at the interface could also play a role. Charge transfer at the interface or changes in the local electric field due to the filler can modify the electronic states of the composite and influence the absorption edge. The blue shift of the optical absorption edge in PVDF with SrTiO_3_/CNT nanofiller highlights the potential for tailoring the material’s optical and electronic properties for various applications [50].

SrTiO_3_/CNTs are added to PVDF polymer films, which results in improved optical characteristics by reducing transmittance, as shown in Figure 4b. For applications, including optical devices that need controlled light transmission, this drop in transmittance is favorable [51]. The inclusion of nanofillers causes a decline in optical transmittance within polymer films. This can be explained by the fact that the polymer and the filler particles form intermolecular complexes, which increase light scattering [52].

### 3.5. Optical Band Gap Investigations

**Figure 5:**
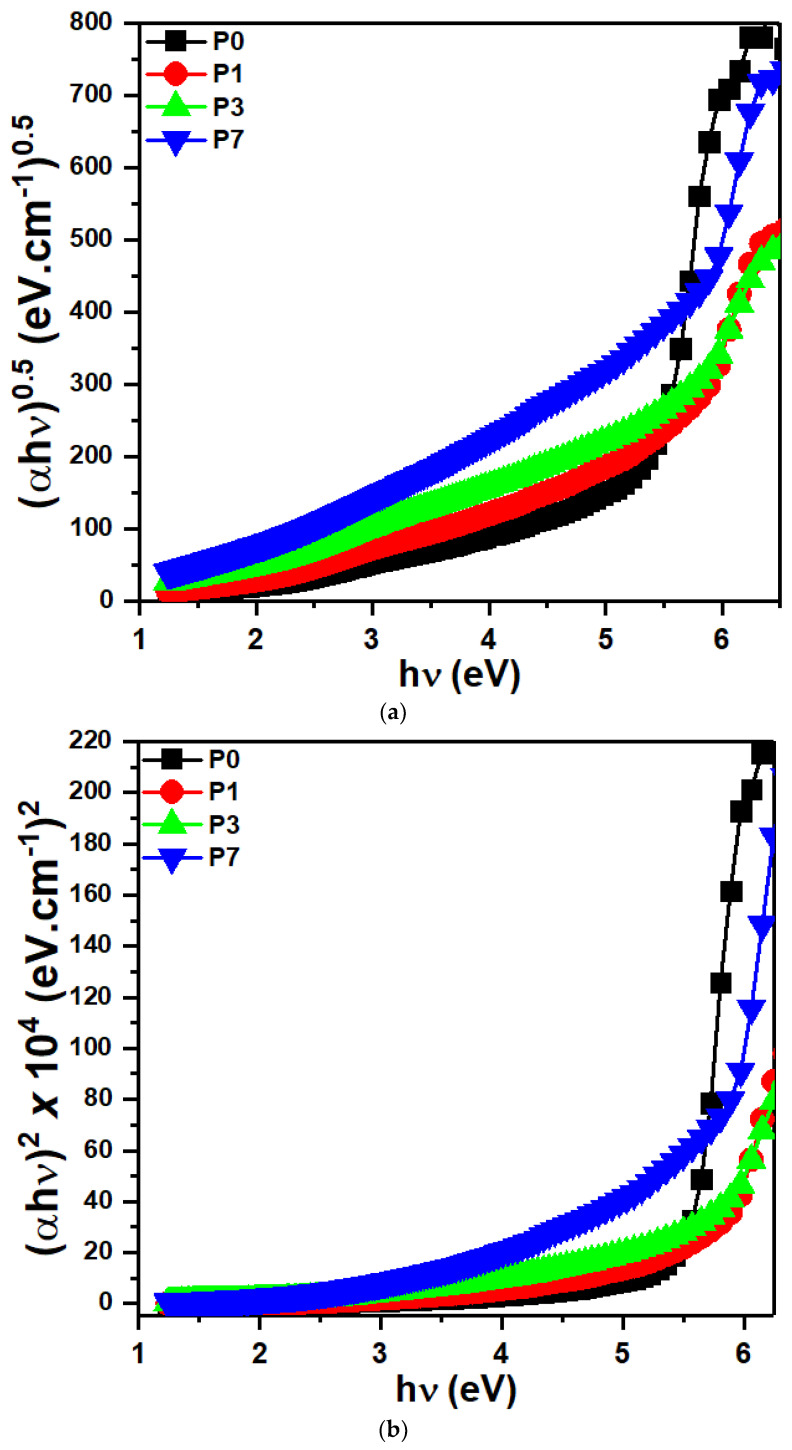
(**a**) Plots of (αhυ)^0.5^ vs. hυ for the PVDF/SrTiO_3_/CNTs films. (**b**) Plots of (αhυ)^2^ vs. hυ for the PVDF/SrTiO_3_/CNTs films.

Polymer film characterization often relies on the Tauc equation to analyze the optical band gap and internal electronic transitions. The intersection of the extrapolated linear portion of a Tauc plot with the energy axis yields the optical band gap [53,54]:

(2) $$\alpha h\upsilon =B{\left(hv-E_{g}\right)}^{n}$$

where *hv* is the energy of the incident photons, *B* is a constant, and α is the absorption coefficient. Direct allowed transitions occur when n has a value of 0.5, while indirect allowed transitions require n to be 2. Figure 5a,b present a method for estimating the bandgap energy. This involves extending the linear region observed at the onset of the absorption edge back to the energy axis, where the intercept provides the desired value.

Table 1 presents the obtained band gaps (E_g(ind)_ and E_g(dir)_). FTIR analysis demonstrates that SrTiO_3_/CNTs nanofillers promote the development of molecular complexes between the polymer matrix and the filler [51], as evidenced by the band gaps listed in Table 1. The observation that the optical band gap of PVDF polymer films increases after incorporating 0.1 wt% SrTiO_3_/CNT nanofiller is a fascinating finding with potential implications for optoelectronic applications.

The presence of SrTiO_3_/CNTs disrupts chain packing and π-π* conjugation within the PVDF matrix, effectively decreasing the number of available excited states and widening the band gap. A wider band gap can be beneficial for certain optoelectronic applications like light-emitting diodes (LEDs) or photodetectors. At higher doping levels, the concentration of SrTiO_3_/CNT at the interface with PVDF could increase significantly. This might lead to the formation of new electronic states within the band gap due to strong interfacial interactions. These states could act as intermediate levels for electronic transitions, effectively narrowing the band gap compared to a situation with lower filler content [55].

### 3.6. Linear Refractive Index

**Figure 6:**
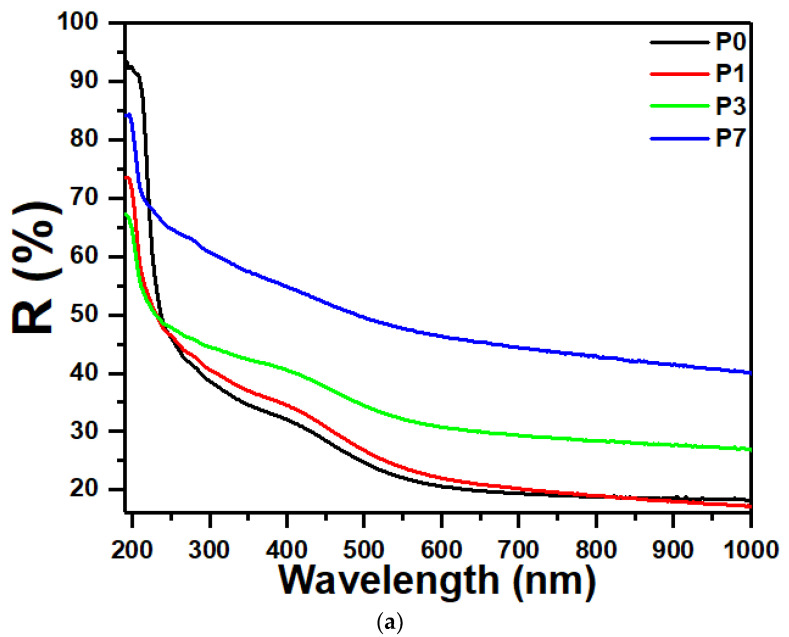

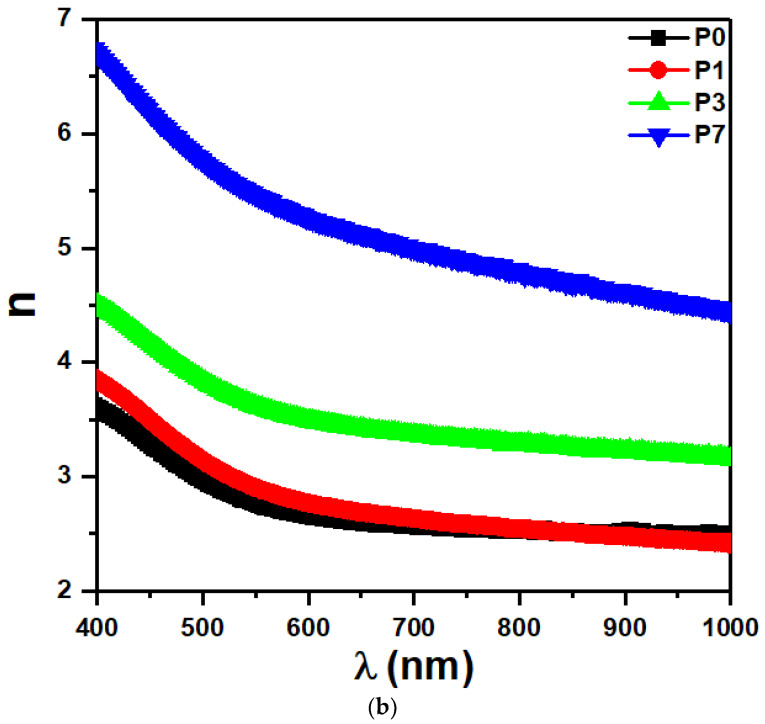
(**a**) Plots of reflectance (R) vs. wavelength for the PVDF/SrTiO_3_/CNTs films. (**b**) Plots of refractive index vs. wavelength for the PVDF/SrTiO_3_/CNTs films.

The investigation of the refractive index of polymer films presents numerous advantages. High refractive index polymer films have also been investigated for their optical and electrical performance in optoelectronic applications. Several factors, including the type of molecules making up the polymer, the inclusion of fillers, the film’s overall depth, and the stretching of a polymer film, control different interactions with light, as measured by its refractive index [56]. Reflectance quantifies the proportion of incident light that a surface reflects. Figure 6a displays the spectral reflectance (variation of reflectance with wavelength) of SrTiO_3_/CNTs samples with different compositions. The optical reflectance of the polymer films increased upon increasing the nanofiller content. This is because the nanofillers scatter light, which reflects off the surface of the film.

The following equation is used to find the relationship between reflectance (*R*) and refractive index (*n*) [35,56]:

(3) $$n=\left(\frac{1+R}{1-R}\right)+\;\sqrt{\frac{4R}{{\left(1-R\right)}^{2}}-k^{2}},$$

where (*k* =αλ/4π) signifies the extinction coefficient. The refractive index directly increases with the SrTiO_3_/CNTs content (Figure 6b). The addition of SrTiO_3_/CNTs might increase the overall density and packing density of the PVDF matrix. This can lead to a higher concentration of polarizable units within the material, leading to a stronger interaction with the electric field of the light wave and, consequently, a higher refractive index. Strong interfacial interactions between the SrTiO_3_/CNTs and the PVDF chains can induce additional polarization effects at the interface. These interfacial polarizations contribute to the overall response of the material to the light wave, further increasing the refractive index. High-refractive-index polymer films have the potential to revolutionize a variety of optical applications due to their unique properties. These films, made by combining small SrTiO_3_/CNTs into a large cluster, can be used to create optical coatings, anti-reflection coatings, and even optical devices. Their enhanced refractive index makes them ideal for applications such as reflectors and nanophotonic systems [57,58].

Wemple and DiDomenico’s single oscillator model simplifies calculating refractive index by estimating key material parameters like oscillator and dispersion energies [59]. Accordingly, the refractive dispersion of a material can be attributed to the contributions of its single-oscillator energy (*E*_0_) and dispersion energy (*E_d_*), both of which are determined within the framework of their single-oscillator model [60].

(4) $${\left(n^{2}-1\right)}^{-1}=\frac{E_{0}}{E_{d}}-\frac{1}{E_{0}E_{d}}\;{\left(h\upsilon \right)}^{2}$$

**Figure 7:**
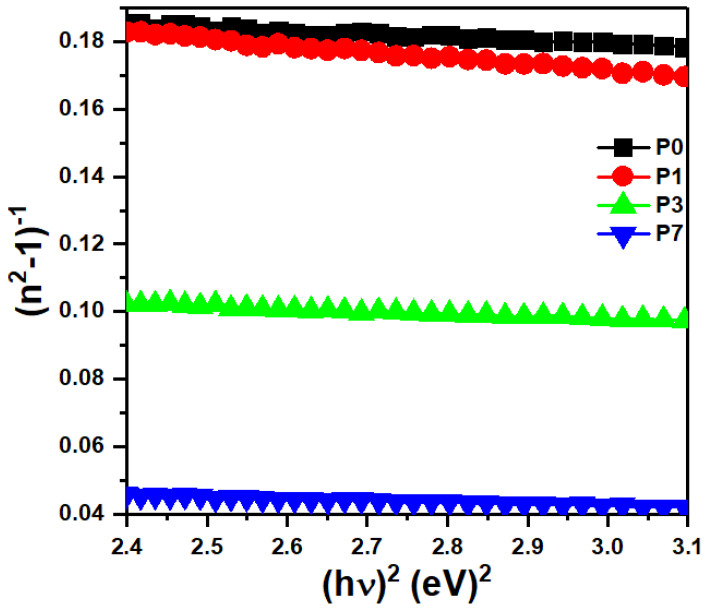
Plots of (n^2^ − 1)^−1^ vs. ${\left(h\upsilon \right)}^{2}$ for the PVDF/SrTiO_3_/CNTs films.

In Figure 7, the slopes and intercepts of the (n^2^ − 1)^−1^ versus (*hν*)^2^ plots were analyzed to extract the values of *E*_0_ and *E_d_*. As the concentration of SrTiO_3_/CNTs nanofiller increased (see Table 1), E_0_ exhibited a noticeable shift from 4.77 eV to 3.70 eV, while E_d_ displayed a wider range of variation, changing from 23.08 eV to 63.63 eV. The presence of 0.1 wt% SrTiO_3_/CNTs in a polymer film leads to the denser packing of the polymer chains due to their alignment. This closer proximity enhances intermolecular interactions, resulting in a rise in interchain attraction forces [61]. In thin polymer films, the long-range van der Waals forces, also known as dispersion forces, can amplify the natural vibrations of the polymer chains due to thermal energy. This increased chain mobility further contributes to the observed increase in interchain interactions when incorporating 0.1 wt% SrTiO_3_/CNTs, which likely promotes chain alignment [62]. With an increasing SrTiO_3_/CNTs content, the interaction between the filler and the PVDF matrix might change. At higher loadings, the interfacial region might become saturated, leading to weaker interactions and a reduced contribution to the overall dispersion energy. Additionally, interfaces between different materials can create regions where electrons redistribute, leading to charge transfer. In this case, charge transfer could occur between PVDF and SrTiO_3_/CNTs at their interface. This charge transfer modifies the electrical properties of the composite, potentially affecting its response to an electric field [63].

The single oscillator model provided by Wemple and DiDomenico at zero photon energy (*hν* = 0) was used to calculate the static refractive index (*n*_0_) of a polymer as follows [64]:

(5) $${n_{0}}^{2}=\left(1+\frac{E_{d}}{E_{0}}\right)$$

The incorporation of SrTiO_3_/CNTs nanofiller resulted in a significant enhancement in the refractive index (*n*_0_) of the material, with values ranging from 2.42 to 4.27. This paves the way for the development of high-performance optoelectronic devices such as advanced displays, light-emitting diodes, and plastic lenses [65]. The addition of a SrTiO_3_/CNT nanofiller presents a promising approach for improving the refractive index of polymer films, opening exciting possibilities for various optical applications. A higher refractive index allows for the tighter focusing of light, leading to smaller and more efficient microlenses. This is crucial for miniaturization in optical devices. Enhanced light manipulation within the polymer film can improve the sensitivity and resolution of image sensors. This could benefit areas like medical imaging and surveillance. Furthermore, controlling the refractive index of the emitting layer in OLEDs can optimize light extraction and improve device efficiency and brightness [66].

### 3.7. Nonlinear Optical Parameters

Linear susceptibility describes the basic interactions with light, like refraction and reflection. Nonlinear susceptibility, on the other hand, governs more complex interactions, like harmonic generation and second-order processes, which are critical for nonlinear optical devices. The magnitude and nature of these susceptibilities determine the film’s suitability for various applications. For example, high linear susceptibility is desirable for optical data storage to achieve strong signal modulation, while high nonlinear susceptibility is crucial for devices like optical switches and frequency converters [67]. The linear optical susceptibility (*χ*^(1)^) and third-order nonlinear optical susceptibility (*χ*^(3)^) of polymer films can be calculated using the following relations [68]:

(6) $$\chi^{\left(1\right)}=\frac{E_{d}/E_{0}}{4\pi },\;x^{\left(3\right)}=6.82\times 10^{-15}{\left(E_{d}/E_{0}\right)}^{4}$$

Table 2 presents estimations of the first-order (*χ*^(1)^) and third-order (*χ*^(3)^) nonlinear optical susceptibilities in PVDF/SrTiO_3_/CNTs polymer films. Nanofillers, due to their high aspect ratio and dielectric contrast with the polymer matrix, can concentrate the electric field at the interface. This localized field enhancement leads to a stronger interaction between light and the material, consequently amplifying the nonlinear optical response. This phenomenon finds applications in devices like optical modulators and sensors, where a small change in the input light intensity can lead to a significant change in the output. Moreover, when nanofillers interact with polymer molecules, they can create new energy levels within the bandgap of the composite material. These new levels can participate in virtual transitions induced by the incident light, enhancing the nonlinear optical response [69,70].

The chemical structure of a polymer film directly shapes the polarizability of its molecules, thereby modulating the film’s nonlinear refractive index (*n*_2_) [71]. Polymer films with high NRI can be used to create compact and efficient waveguides for guiding and manipulating light signals. This is crucial for on-chip optical circuits and integrated photonic devices. The nonlinear refractive index enables processes like harmonic generation and parametric amplification, which are essential for generating new wavelengths of light and manipulating their properties. This has applications in areas like spectroscopy, optical imaging, and biophotonics [72]. The nonlinear refractive index (*n*_2_) can be computed using the equation [73,74]:

(7) $$n_{2}=\frac{12\pi x^{\left(3\right)}}{n_{0}}$$

Table 2 presents the estimated data for *n*_2_. The addition of SrTiO_3_/CNT nanofillers enhances the polarizability of the polymer molecules, resulting in a higher nonlinear refractive index. The improvement of the nonlinear refractive index of polymer films is a rapidly evolving field with vast potential. The unique combination of material properties and diverse applications makes it an exciting area to watch, promising breakthroughs in photonics, communication, and various other fields. By improving the nonlinear refractive index, it is possible to develop polymer films with even better optical properties for a variety of applications. The high nonlinear refractive index could be used to improve the efficiency of photovoltaic cells by concentrating light on the active area of the cell. The increased nonlinear refractive index leads to stronger second harmonic generation (SHG) and other nonlinear optical effects. This could be used to manipulate the direction and distribution of light within the solar cell, potentially leading to more efficient light harvesting and improved conversion efficiency. Furthermore, CNTs within the nanofillers act as light scatterers, trapping light within the active layer of the solar cell and increasing the path length for photon absorption. This could also contribute to enhanced light harvesting.

The band gap, refractive index, and optical susceptibility of PVDF/SrTiO_3_/CNTS nanocomposite films were compared to previous work. PVDF/SrTiO_3_/CNTS exhibits a larger band gap (5.53 eV) compared to most entries in Table 3. The *χ*^(3)^ value (597.7 × 10^−12^ esu) of PVDF/SrTiO_3_/CNTS is comparable or lower than other entries. The n_2_ value (528.09 × 10^−11^ esu) of PVDF/SrTiO_3_/CNTS falls within the range of other entries. The results demonstrate the potential of PVDF/SrTiO_3_/CNTS nanocomposite films as functional materials with competitive optical properties.

## 4. Conclusions

The present work prepared PVDF/SrTiO_3_/CNTS polymer nanocomposite films. The polymer films were prepared via the solution casting technique at doping ratios of both nanofillers of 0.0, 0.1, 0.3, and 0.7 wt%. The structure properties were investigated by XRD, FTIR, and SEM microscopy analysis. The crystal structure of SrTiO_3_ was cubic, and the XRD diffraction peaks located at 2θ = 20.17° and 39.4° are related to the α phase of the (110) and (002) planes. The main two peaks located at 2θ = 20.02° and 38.9° for the PVDF sample containing 0.1 wt% SrTiO_3_/CNT shifted to higher positions (2θ = 20.26° and 39°, respectively) when increasing the content of SrTiO_3_/CNT to 0.3 wt%. The SEM surface morphology analysis showed that the size and shape of the spherulites were affected by the doping rate of the nanofiller. The addition of a nanofiller resulted in modifications in the electronic structure of the polymer, leading to a decrease in the energy gap. The obtained values of E_0_ showed a variation (4.77–3.70 eV), while E_d_ changed (23.08–63.63 eV) upon increasing the concentration of SrTiO_3_/CNTs nanofiller. The estimated values of static refractive index (n_0_) showed an increase (2.42–4.27) upon increasing the percentage of SrTiO_3_/CNTs nanofiller. The addition of SrTiO_3_/CNT nanofillers increased the polarizability of the polymer molecules and, hence, the nonlinear refractive index. The improvement of the nonlinear refractive index of polymer films is a promising area of application.

## Figures and Tables

**Table 1:** Optical parameters of PVDF/SrTiO_3_/CNTs polymer films.

SrTiO_3_/CNTs (wt%)	E_g(ind)_ (eV)	E_g(dir)_ (eV)	E_0_ (eV)	E_d_ (eV)	n_0_
0.0	5.27	5.56	4.77	23.08	2.42
0.1	5.50	5.70	3.48	15.29	2.32
0.3	5.40	5.60	4.06	33.95	3.06
0.7	5.30	5.53	3.70	63.63	4.27

**Table 2:** Dispersion parameters PVDF/SrTiO_3_/CNTs polymer films.

SrTiO_3_/CNTs (wt%)	χ^(1)^ (esu)	χ^(3)^ × 10^−12^ (esu)	n_2_ × 10^−11^ (esu)
0.0	0.38	3.73	5.82
0.1	0.35	2.53	4.12
0.3	0.66	33.26	40.98
0.7	1.37	597.70	528.09

**Table 3:** The band gap, refractive index, and optical susceptibility of PVDF/SrTiO_3_/CNTS nanocomposite films compared to previous work.

Polymer Nanocomposite	Direct Band Gap (eV)	Indirect Band Gap (eV)	χ^(3)^ (esu)	n_2_ (esu)	Ref.
PVDF/CaFe_2_O_4_	5.15	4.18	870 × 10^−9^	3.17 × 10^−6^	[36]
PVDF/MoO_3_/g-C_3_N_4_	4.5	4.0	2.5 × 10^−10^	2.5 × 10^−9^	[57]
PVDF/ZnO	4.95	3.35	-	-	[31]
PVDF/RGO	4.3	3.2	-	-	[75]
PVDF/RGO	-	-	12.96 × 10^−11^	6.27 × 10^−10^	[76]
PVDF/Li_4_Ti_5_O_12_	4.858	1.005	1.0 × 10^−6^	6.0 × 10^−6^	[77]
PVDF/SrTiO_3_/CNTS	5.53	5.30	597.7 × 10^−12^	528.09 *×* 10^−11^	This work

## Data Availability

The raw data supporting the conclusions of this article will be made available by the authors on request.
